# Two natural toughening strategies may inspire sustainable structures

**DOI:** 10.1038/s41598-023-47574-y

**Published:** 2023-11-21

**Authors:** Israel Greenfeld, H. Daniel Wagner

**Affiliations:** Department of Molecular Chemistry and Materials Science, Weizmann Institute of Science, 76100 Rehovot, Israel

**Keywords:** Composites, Mechanical properties, Bioinspired materials

## Abstract

Contemporary designs of engineering structures strive to minimize the use of material in order to reduce cost and weight. However, the approach taken by focusing on materials selection and on the design of the exterior shape of structures has reached its limits. By contrast, nature implements bottom-up designs based on a multiple-level hierarchy, spanning from nanoscale to macroscale, which evolved over millions of years in an environmentally sustainable manner given limited resources. Natural structures often appear as laminates in wood, bone, plants, exoskeletons, etc., and employ elaborate micro-structural mechanisms to generate simultaneous strength and toughness. One such mechanism, observed in the scorpion cuticle and in the sponge spicule, is the grading (gradual change) of properties like layers thickness, stiffness, strength and toughness. We show that grading is a biological design tradeoff, which optimizes the use of material to enhance survival traits such as endurance against impending detrimental cracks. We found that such design, when applied in a more vulnerable direction of the laminate, has the potential to restrain propagation of hazardous cracks by deflecting or bifurcating them. This is achieved by shifting material from non-critical regions to more critical regions, making the design sustainable in the sense of efficient use of building resources. We investigate how such a mechanism functions in nature and how it can be implemented in synthetic structures, by means of a generic analytical model for crack deflection in a general laminate. Such a mechanical model may help optimize the design of bioinspired structures for specific applications and, eventually, reduce material waste.

## Introduction

Principles and strategies found in nature may be adapted to the design and development of sustainable products, systems, and technologies. Taking inspiration from nature, important challenges in understanding novel synthetic material designs may be addressed, including improved performance, environmental compatibility, longer use, higher reliability, and long-term sustainability. This approach is based on the premise that natural structures have evolved in an environmentally sustainable manner, maintaining an ecological balance in earth’s natural environment^1^, and achieving desired functionality over millions of years under the constraints of limited resources. The efficiency, resilience, and adaptability of natural organisms can be viewed as a model for human-made systems, to yield products and technologies that have a lower impact on the environment, use resources more efficiently, and have a longer lifespan. The ensuing methodology of sustainable design, generally termed *bioinspired sustainability*, has recently been the subject of several conceptual plans and case studies^2,3^. A recent review article provides important information about advanced bio-based materials and their composites for prospective usage in different high-performance applications^4^.

**Figure 1:**
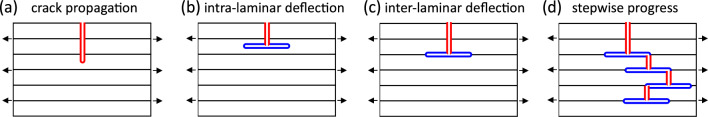
Crack propagation and deflection in a laminate. (**a**) Propagation (Griffith crack). (**b**) Intra-laminar deflection or bifurcation. (**c**) Inter-laminar deflection or bifurcation. (d) Stepwise alternating propagation-deflection progress.

Scientists and engineers are constantly searching for more efficient ways to achieve specific functionalities and performance: a suitable structural design always strives to minimize the use of material in order to reduce cost and weight. However, the design of engineering structures, which usually focuses on materials selection and top-down forming of the external geometry, is by far different from nature. Natural structures, such as wood, bone, plants, and exoskeletons, are typically built bottom-up in multiple-level hierarchy, spanning from nanoscale to macroscale, and separated by hierarchical interfaces which serve as fracture propagation traps^5^. These natural layered composites employ elaborate micro-structural mechanisms to generate simultaneous strength and toughness, often conflicting properties^6^. Such mechanisms involve anisotropy to induce strengthening in desired directions^7^, soft interfaces in ceramic-based composites such as bone to overcome their inherent brittleness by deflecting cracks^8–13^, variable layer thickness in a laminated structure such as the sponge spicule^14–16^, graded stiffness in the scorpion cuticle to encourage specifically localized crack deflection^17^, thin layers which limit the penetration depth of cracks by forcing early deflection^18^ and which become progressively insensitive to flaws at nanoscale^19^. Localized deflection of a crack propagating in a laminate, the subject of this study, is illustrated in Fig. 1.

Natural designs often favor some structural properties over others, as found in our ongoing research on the scorpion cuticle^5,17,20^. The latter consists of layers of helical building blocks (termed Bouligands), the thickness and stiffness of which decrease from the outside surface to the animal inside. Our recent analysis demonstrated that this conformation is more damage tolerant (that is, resistant) against external cuticle defects, and less tolerant against internal defects^17^. When considering the harsh external environment to which a scorpion is exposed, one may indeed expect defects that are more severe on the outside than in the inside. This seems as a design tradeoff, which does not seek to optimize all properties but rather optimizes the use of material to enhance life-saving properties. In other words, this design is sustainable in the sense of efficient use of natural resources.

Importing these concepts into human-made designs is highly challenging, particularly because natural structures are built bottom-up, starting from basic nano-components such as ceramic platelets and chitin, collagen or cellulose filaments, packed into intermediate subunits such as Bouligands, then arranged in a laminated composite (to mention just a few of the hierarchical levels). That said, some natural micro/nano structures and mechanisms, such as tiny building blocks separated by various types of interfaces to slow down or arrest cracks, and grading of properties to deflect cracks, are definitely importable into engineering.

Bioinspired structures and mechanisms, and their potential sustainability benefits, are the subjects of this study. A key to engineering implementation is to acquire a deeper understanding of how these mechanisms function. The nano and micro scales involved render pinpoint measurements impractical or extremely difficult; therefore, our efforts essentially focus on modeling and analysis, which may help optimize the design of bioinspired structures for specific applications and, eventually, reduce material waste. The model presented here predicts the conditions for crack deflection in a general multilayer, multimaterial laminate, specifically with graded layer thickness and stiffness^17^. Whether a crack tends to propagate parallel to itself in a Griffith-like fashion or to bifurcate in a deflected direction has been extensively studied for the bilayer case^21–29^, and bimaterial with uniform layers thickness^30^, but not for the general case of graded stiffness, multilayer (with variable thickness), multimaterial structures; Wagner et al. partially addressed this problem, modeling the stress required for delamination^31,32^, but not the stress required for propagation which is necessary for setting a deflection criterion.

Here, we begin by presenting two very different schemes employed by nature to deflect propagating cracks in brittle structures. The first is based on the layered architecture of the scorpion cuticle, which uses chitin fibers as reinforcement at varying layer densities. The second scheme appears in the layered architecture of the sponge spicule, reinforced by silica. Both are arranged in layers of varying thickness but, as will be seen, are only similar in appearance. We present a multilayer, multimaterial laminate cracking model, based on classical fracture mechanics, which has the merit of simplicity with no loss of physical meaning. The model is applied to these two structural types, demonstrating the different mechanisms by which each scheme deflects and arrests a propagating crack. Lastly, we discuss various laminate arrangements, comprising different combinations of graded layer thickness and stiffness, and examine their theoretical resilience (or damage tolerance) and likely benefits for sustainable engineering structures.

## Two biological structures

**Figure 2:**
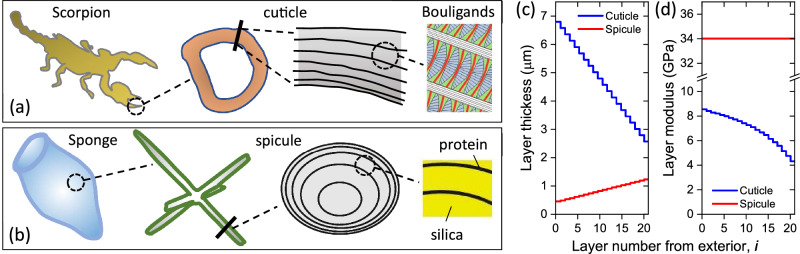
The different structures of two representative biological laminates. (**a**) The scorpion’s cuticle. (**b**) The sponge’s spicule. (**c**) Typical layer thickness variation. (**d**) Typical layer stiffness (modulus) variation.

The scorpion cuticle and the sponge spicule are both stiff and strong laminate structures, which serve as the species main scaffolding elements (Fig. 2a,b). These structures have gone through separate evolutionary paths, and therefore their morphologies are essentially different. The cuticle is built of closely packed layers of Bouligands, anisotropic helical structures consisting of numerous twisted (in-plane rotation) and tilted (out-of-plane rotation) laminae of unidirectional chitin fibers embedded in a proteinaceous matrix^5,17,20,33^. By contrast, the spicule is built of homogeneous isotropic silica layers, separated by silicatein, a soft proteinaceous matrix^14–16,18,34^. Both laminates have varying layer thickness from exterior to interior, decreasing in the cuticle whereas increasing in the spicule (Fig. 2c)^16,17^. The cuticle has, in addition, decreasing stiffness (elastic modulus) from exterior to interior, associated with decreasing chitin fraction due to laminae tilting^16^, whereas the spicule has a practically uniform modulus (Fig. 2d)^34^.

The scorpion and sponge are representative structural types, and are considered here as examples for: (i) A fibrous cuticle-like laminate, consisting of strong and stiff chitin fibers embedded in soft protein, possessing a hierarchical structure which spans from nano to micro scale. The proteinaceous interfaces are hierarchical as well^5^, between neighboring fibers, between laminae, between Bouligand units, and between layers. Thus, crack deflection can occur at any level, between fibers, between laminae, and so on. (ii) A ceramic spicule-like laminate, consisting of hard homogenous bio-silica layers with thin soft proteinaceous interfaces. The silica high stiffness deters crack deflection, deferring it to a protein interface. The differences between these biological structures are summarized in Table 1.

**Table 1:** Comparison between the two biological laminate types.

Property	Scorpion-like (cuticle)	Sponge-like (spicule)
Laminate stiffness	Medium, 4–9 GPa^17^	High, 37 GPa^34^
Morphology	Chitin-fiber laminae, embedded in protein	Bio-silica layers, separated by protein
Layers thickness	Decreasing from exterior, 7–3 μm^17^	Increasing from exterior, 0.5–1.2 μm^16^
Layers stiffness	Decreasing from exterior to interior	Uniform
Soft interfaces	At all hierarchical levels	Between silica layers
Crack deflection	Possible anywhere	preferentially between silica layers

**Figure 3:**
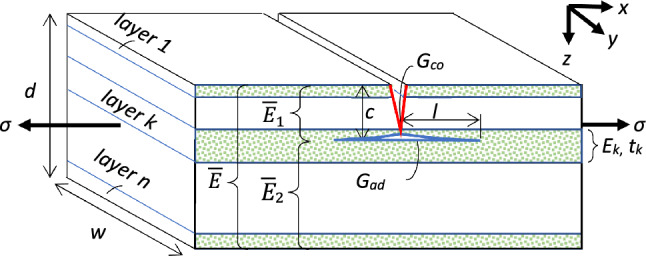
Varying thickness and stiffness laminate—geometry, properties and loading. The stress $\sigma$ applied to the laminate is uniform. A propagating crack of length $c$ (red) penetrates through the laminate into lamina $k$, and may deflect into a delaminating crack of length $l$ (blue). The modulus of lamina $k$ is ${E}_{k}$ and its thickness is ${t}_{k}$. ${\overline{E}}_{1}$ and ${\overline{E}}_{2}$ are the weighted-average moduli of the laminate regions behind and beyond the crack tip, respectively, and the laminate overall weighted-average modulus is $\overline{E}$. ${G}_{co}$ and ${G}_{ad}$ are the cohesive and adhesive fracture energies at the crack tip, respectively (the energies per unit area required to break the bonds at the tip).

The terms Scorpion-like and Sponge-like laminates are used throughout the paper to designate bioinspired synthetic laminates, which have a planar plate geometry with rectangular cross section and parallel layers, as depicted in Fig. 3. The arrangement of the layers, with variable (graded) layer thickness and stiffness, is inspired by the layer structure in the cuticle and spicule. The actual biological examples have an overall circular geometry, but locally their layers are nearly flat and may be roughly approximated by the plate geometry. That said, it is not our intention to provide accurate modeling for the cuticle and spicule, but rather to demonstrate the benefit of layer grading in synthetic laminates.

## Crack deflection condition

Deflection or bifurcation detract a propagating crack from deepening and causing a catastrophic failure, and are therefore beneficial for the laminate toughness and reliability. The applied external stress may result either in cohesive failure at the crack tip and further propagation, or in adhesive failure at an interface and delamination (Fig. 3). Thus, the condition for crack deflection is:

1 $${\sigma }_{ad}<{\sigma }_{co}$$

when the stress causing an adhesive failure, ${\sigma }_{ad}$, becomes lower than the stress causing a cohesive failure, ${\sigma }_{co}$. Expressions for these stresses are derived by applying classic fracture mechanics to a general multilayer, multimaterial laminate^17^.

A crack will propagate when its growth releases stored elastic energy in the region behind the crack tip, sufficient to break the chemical bonds across the crack. Using fracture mechanics, the stress causing further propagation (cohesive failure) of a crack $c$ is given by (plane stress condition)^17^:

2 $${\sigma }_{co}=\sqrt{\frac{{G}_{co}\overline{E}}{\pi c}}\sqrt{\frac{\overline{E}}{{\overline{E}}_{1}}}$$

where the laminate geometry, properties and loading are defined in Fig. 3. The first factor is the classic Griffith term, whereas the second is a correction term reflecting the inhomogeneity of the laminate. Thus, when the average stiffness in the laminate region behind the crack tip, ${\overline{E}}_{1}$, is higher than that of the entire laminate, $\overline{E}$, the cohesive fracture stress will be lower, and vice versa.

Similarly, delamination will progress when its growth releases stored elastic energy in the region behind the crack tip (denoted by ${\overline{E}}_{1}$ in Fig. 3), sufficient to break the chemical bonds across the interface. In this case, portions of the released energy are gained by the region beyond the crack tip (denoted by ${\overline{E}}_{2}$ in Fig. 3), whose section of length $2l$ elongates, and by the work invested in laminate stretching (the applied stress times the laminate edge displacement). The stress causing delamination (adhesive failure) is given by^17^:

3 $${\sigma }_{ad}=\sqrt{\frac{{2G}_{ad}\overline{E}}{c}\left(1-\frac{c}{d}\right)}\sqrt{\frac{{\overline{E}}_{2}}{{\overline{E}}_{1}}}$$

The first factor is a Griffith-like term, whereas the second is an inhomogeneity correction term. Thus, when the average stiffness in the laminate region behind the crack tip, ${\overline{E}}_{1}$, is higher than that of the region beyond the tip, ${\overline{E}}_{2}$, the adhesive fracture stress will be lower, and vice versa.

Both the cohesive and adhesive fracture stresses decrease as the crack $c$ deepens, but the adhesive stress decreases faster because of the term $1-c/d$ and eventually becomes smaller than the cohesive stress, enabling deflection. Substituting the stresses from Eqs. (2) and (3) in Eq. (1) and rearranging, the energy-based condition for crack deflection from cohesive failure to adhesive failure is obtained^17^:

4 $$\frac{{G}_{ad}}{{G}_{co}}<\frac{1}{4\pi \left(1-c/d\right)}\frac{\overline{E}}{{\overline{E}}_{2}}$$

where, to account for adhesive crack initiation, a factor of $\sqrt{2}$ was applied to the adhesive stress^17,21,35^. Here as well, the stiffness ratio term is an inhomogeneity correction factor, such that when the stiffness in the laminate region beyond the crack tip, ${\overline{E}}_{2}$, is lower than that of the entire laminate, $\overline{E}$, the likelihood of crack deflection will be higher, and vice versa. Likewise, considering the fracture energy ratio term, when the adhesive fracture energy at the crack tip is low with respect to the cohesive energy at the crack tip, the likelihood of crack deflection will be higher, and vice versa. For a homogenous laminate and small cracks, deflection will occur when the energy ratio is ${G}_{ad}/{G}_{co}<{\left(4\pi \right)}^{-1}$. In an inhomogeneous laminate, the variables ${G}_{ad}$, ${G}_{co}$, ${\overline{E}}_{1}$ and ${\overline{E}}_{2}$ are functions of the crack depth $c$, as the fracture energy and stiffness properties change from layer to layer.

In the extreme case when $c\to d$, the condition seems to predict that deflection always occurs, regardless of the other parameters; however, in such geometry the stress intensity factor near the crack tip rises sharply (see AFGROW Handbook)^36^ above the classic Griffith solution, and the deflection condition should be adjusted accordingly. The finite element analysis (FEA) in Appendix 2 shows that the stress intensity factor near the tip of a deep propagating crack diverges faster than near the tip of a delaminating crack, rendering deflection unlikely. For smaller cracks, this geometric correction is nearly the same for both crack types, and its effect is cancelled in the deflection condition of Eq. (4).

The weighted-average moduli in Eqs. (2), (3) and (4) are calculated by summing up the moduli of the relevant laminae, taking into consideration the location of the crack tip inside a lamina^17^:

5 $$\begin{aligned}{ }\overline{E}&=\frac{1}{d}\sum_{i=1}^{n}{E}_{i}{t}_{i}\\ {\overline{E}}_{1}&=\frac{1}{c}\left[\sum_{i=1}^{k}{E}_{i}{t}_{i}-{E}_{k}\left(\sum_{i=1}^{k}{t}_{i}-c\right)\right]\\ {\overline{E}}_{2}&=\frac{1}{d-c}\left[\sum_{i=k}^{n}{E}_{i}{t}_{i}-{E}_{k}\left(\sum_{i=k}^{n}{t}_{i}-d+c\right)\right]\end{aligned}$$

where the various parameters are defined in Fig. 3.

The described model applies to a flat laminate under unidirectional tensile loading, in the presence of a penetrating crack whose surface is planar and perpendicular to the load direction. The model provides a proof of concept for the grading of properties like layer thickness and stiffness. In practice, typical biological structures such as those shown in Fig. 2, as well as some engineering designs, have complex convoluted shapes, and are subjected to complex loading conditions involving tension and bending in multiple directions. However, the modeling is based on the universal concepts of fracture mechanics, which balances the net released and gained elastic energy of the overall structure against the crack fracture energy (a material property), determining whether a crack will continue propagating, stop or deflect. Grading changes the stress distribution in a laminate, and consequently the amount of released and gained elastic energies, compared to a uniform laminate. Evidently, the cross-sectional geometry of a specific structure, and the direction of an initial crack, affect the stress distribution as well, and should therefore be analyzed specifically for each geometry, along the same theoretical approach. The specifics of such modeling may differ from the model described above, to reflect different geometries, but the basic dependence on the average moduli behind and beyond the crack tip should apply.

Furthermore, the model was expanded to bending loading of a planar laminate (for details see Appendix 1: Deflection condition in bending), demonstrating the applicability of the grading approach in a different loading type. With regard to the scorpion cuticle and the sponge spicule, bending along their longitudinal axis is common in their living conditions. This was tested in bending fracture experiments of cuticles and spicules, which demonstrated the inherent mechanisms of crack deflection in their layered structure (see details in Section "Experimental evidence").

## Toughening strategies

Using the deflection model, the two biological structures may be used as examples for demonstrating two distinctly different toughening strategies: (i) graded layer stiffness and thickness in a scorpion-like structure, representative of fiber-reinforced composites, and (ii) graded layer thickness in a sponge-like structure, representative of ceramic-reinforced composites. Both strategies, when applied to the laminate in specified directions, may trigger early deflection of a propagating crack, preventing immediate catastrophic failure.

To begin with, we need to assess the dependence of the variables ${\overline{E}}_{1}$, ${\overline{E}}_{2}$, ${G}_{ad}$ and ${G}_{co}$ (Eqs. (2)–(4) in the deflection model) on the crack depth $c$. The dependence of ${\overline{E}}_{1}$ and ${\overline{E}}_{2}$ on $c$ is simply calculated by Eqs. (5), given the modulus and thickness of each layer. The dependence of the fracture energies ${G}_{ad}$ and ${G}_{co}$ on $c$ can be assessed by applying the following simplifications: (i) The adhesive energy in a sponge-like laminate is constant in the interfaces between silica layers, equal to that of the protein matrix, and constant inside the silica layers, equal to the cohesive energy of silica (assumed isotropic); the adhesive fracture energy in a scorpion-like laminate is constant, equal to that of the protein matrix. (ii) The cohesive fracture energy in a sponge-like laminate is constant, equal to that of the silica; the cohesive fracture energy in a scorpion-like laminate may be assessed by invoking the proportionality between the fracture energy and the tensile modulus in brittle materials^17,37^, that is:

6 $${G}_{co}\approx \frac{{\overline{G}}_{co}}{\overline{E}}E$$

where ${\overline{G}}_{co}$ is the laminate overall cohesive fracture energy (which may be estimated by fracture toughness tests of the whole laminate), and $E$ is the laminate modulus at the crack tip (not an average). At layer $k$, $E={E}_{k}$, and ${G}_{co}={{G}_{co}}_{k}$ can then be calculated using this equation.

**Figure 4:**
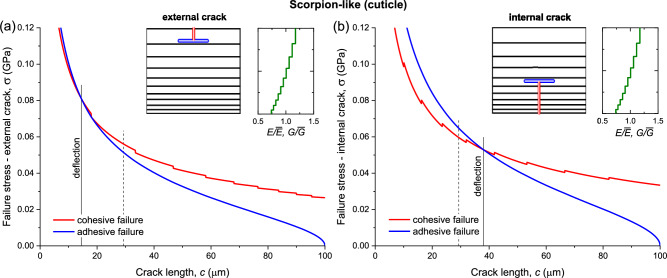
Cohesive and adhesive failure stresses of the scorpion-like (cuticle) structure. (**a**) External crack (decreasing modulus and thickness). (**b**) Internal crack (increasing modulus and thickness). The insets show the modulus and fracture energy grading, layers grading, and calculated location of deflection. The solid vertical lines mark the position where the adhesive stress becomes lower than the cohesive stress, resulting in crack deflection. The dashed vertical lines mark the deflection position in a laminate without any grading (uniform layers thickness and stiffness). Data from Table 2.

**Figure 5:**
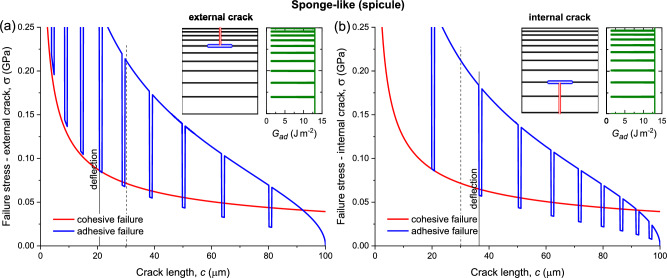
Cohesive and adhesive failure stresses of the sponge-like (spicule) structure. (**a**) External crack (increasing thickness). (**b**) Internal crack (decreasing thickness). The insets show the adhesive fracture energy fluctuation between silica (vertical line, 13 J/m^2^) and protein (horizontal ‘negative’ peaks, 1.3 J/m^2^), layers thickness grading, and calculated location of deflection. The solid vertical lines mark the position where the adhesive stress becomes lower than the cohesive stress, resulting in crack deflection. The dashed vertical lines mark the deflection position in a laminate without grading (uniform layers thickness and stiffness). Data from Table 2.

The material properties used in the following examples are summarized in Table 2. Based on these data, the calculated cohesive and adhesive failure stresses in the scorpion-like cuticle structure and the sponge-like spicule structure, as functions of the crack length, are presented in Figs. 4 and 5, respectively. Thickness and stiffness gradings were implemented by using geometric series with ${r}_{t}$ and ${r}_{B}$ the ratio between adjacent terms (defined in Table 2), respectively, while keeping the laminate overall thickness and average stiffness constant to allow comparison between cases. Other types of grading, such as linear variation in thickness and stiffness, are possible as well.

**Table 2:** Material properties used in the examples of the two biological laminate types.

Property		Scorpion-like (cuticle)	Sponge-like (spicule)
Laminate thickness (μm)	$d$	100	100
Number of layers	$n$	10	10
Average stiffness (GPa)	$\overline{E}$	8^a^	37^f^
Average cohesive energy (J/m^2^)	${\overline{G}}_{co}$	37^b^	13^g^
Adhesive fracture energy—layer (J/m^2^)	${G}_{ad}$	4.2^c^	13^g^
Adhesive fracture energy—interface (J/m^2^)	${G}_{ad}$	4.2^c^	1.3^h^
Thickness grading factor (from exterior)^d^	${r}_{t}$	0.86	1.2
Stiffness grading factor (from exterior)^d^	${r}_{E}$	0.95^e^	1

The values in this table are adapted from Section "Two biological structures", and modified for clarity.

^a^Based on scorpion endocuticle nanoindentation tests ($\overline{E}=7.3-8.5$ GPa)^38^.

^b^Based on measurements of the modulus and fracture energy of the crab exoskeleton ($E=15$ GPa, $G=70$ J/m^2^)^39^, adjusted for the lower modulus of the cuticle.

^c^Using ${\overline{G}}_{co}$, adjusted by the ratio between the moduli of the protein matrix ($\le 1$ GPa)^20^ and $\overline{E}$.

^d^Using geometric series with $r$ the ratio between adjacent terms, ${r}_{t}={t}_{i}/{t}_{i-1}$ and ${r}_{E}={E}_{i}/{E}_{i-1}$ ($i=2..n$), while keeping the laminate overall thickness and average stiffness constant.

^e^The cohesive fracture energy grading factor is equal to ${r}_{E}$, based on Eq. (6).

^f^Based on nanoindentation tests^34^.

^g^Based on silica data^40^: $E=72-73.4$ GPa, ${K}_{\mathrm{Ic}}=0.85-1.15$ MPa m^0^^.5^, converted to energy by ${\overline{G}}_{co}={K}_{\mathrm{Ic}}^{2}/\overline{E}$. The adhesive fracture energy of silica is assumed to be the same as the cohesive energy, ${G}_{ad}={\overline{G}}_{co}.$

^h^Assumption, about 30% of ${G}_{ad}$ of the cuticle protein matrix.

The analysis of a scorpion-like structure demonstrates that a crack propagating in the direction of a decreasing modulus tends to deflect at an early stage (Fig. 4a, $c=14.5$ μm), in contrast to a crack propagating in the opposite direction (Fig. 4b, $c=38.0$ μm). This difference is further augmented if the thickness grading is removed ($c=13.3$ μm and $c=40.0$ μm, respectively). Crack deflection in a reference laminate without any grading occurs at $c=29.3$ μm (dashed vertical lines), demonstrating the significant advantage of a decreasing modulus over an increasing modulus. The deflection generally occurs within the bulk of a layer (Fig. 4a,b insets).

The analysis of a sponge-like structure further demonstrates that a crack propagating in the direction of an increasing thickness tends to deflect at an earlier stage (Fig. 5a, $c=20.7$ μm), compared to a crack propagating in the opposite direction (Fig. 5b, $c=36.5$ μm). Crack deflection in a reference laminate without grading occurs at $c=30.1$ μm (dashed vertical lines), showing the advantage of an increasing thickness over a decreasing thickness. However, the dependence on layer thickness is sensitive to the ratio between the adhesive and cohesive fracture energies, sometimes causing a reversal of the deflection condition (see Section "Experimental evidence"). The deflection typically occurs at the protein interface between silica layers (Fig. 5a,b insets).

How do these very different toughening strategies work? In other words, how is early deflection encouraged by each laminate type? In the decreasing modulus case (scorpion), the rates of change of the stored and gained elastic energies with a growing crack are such that when the crack is entering a more compliant medium, the likelihood of deflection is higher. This can be seen in the deflection condition (Eq. (4)), when the average modulus of the region beyond the crack tip, ${\overline{E}}_{2}$, is low (that is, compliant). In the increasing thickness case (sponge), this effect is not present as the modulus is uniform, and the deflection condition may be rewritten by substituting $\overline{E}={\overline{E}}_{2}$ in Eq. (4) and rearranging:

7 $$c>\left(1-\frac{1}{4\pi {G}_{ad}/{G}_{co}}\right)d$$

If the protein interfaces were not present, ${G}_{ad}={G}_{co}$ (the silica fracture energy is isotropic), resulting in deflection at $c>92.1$ μm, a detrimental brittle failure characteristic of silica. However, because of the large difference in adhesive fracture energies between the silica and protein, the fracture energy ratio, ${G}_{ad}/{G}_{co}$, drops abruptly at an interface, increasing the likelihood of deflection. Such deflection depends critically on the specific position of a nearby interface; if the next interface is farther away, the adhesive stress negative peak might not cross the cohesive stress curve, and deflection might be deferred. Such occurrence can adversely reverse the deflection condition, as mentioned above. On the other hand, the stress crossings of the successively repeating adhesive negative peaks imply a possible repetitive transition from propagation to delamination and back (illustrated in Fig. 1d), resulting in the stepwise fracture observed in spicule experiments^41^. Note that Eq. (7) applies also to a uniform laminate without any grading.

## Experimental evidence

The proposed crack deflection model offers the advantage of exploring toughening strategies under various loading scenarios, laminate configurations, and material properties. Experimental analysis of the scorpion's cuticle and the sponge’s spicule structures at the nano and micro scales to monitor crack evolution across layers is a highly challenging task, and it falls outside the scope of this study. Nevertheless, prior studies conducted macroscale mechanical tests that lend support to the model.

Nanoindentation tests were carried out for both the cuticle and spicule^34,38,41^. In these tests a diamond-tipped nanoindenter was used to press against the sample, enabling the calculation of modulus and hardness from force–displacement curves and dent depth at maximum force. The cuticle results indicated modulus in the range of $E=7.3-8.5$ GPa (for dry samples), in line with the modulus profile presented in Fig. 2d and the values used in our simulation of a cuticle-like structure (Table 1)^38^. These measurements represent averages obtained from 30 random points across the endocuticle. The spicule results indicated a modulus of 37 GPa for the bio-silica and 0.7 GPa for the protein interface, obtained by modulus mapping technique combined with reverse finite element analysis^34^. These values were used in our simulation of the spicule-like structure (Table 1).

Quasi-static three-point bending tests were conducted on the cuticle to determine flexural modulus, stiffness, strength, and toughness. The measured moduli were in the range of $E=7.3-11.1$ GPa (for dry samples)^33^, comparable to the nanoindentation results, considering the different testing method. The observed experimental fracture patterns of the cuticle reveal surface cracks, as well as delamination cracks occurring at a typical relative crack length of $c/d\cong 0.2$^33^, confirming that deflection indeed takes place relatively close to the external boundary. This result is similar to the prediction of the deflection model, $c/d\cong 0.15$ (Fig. 4a). The fracture patterns exhibit characteristic diffuse damage, featuring multiple randomly distributed cracks across a substantial region. This observation is implied by the tangency of the cohesive and adhesive fracture stresses over a wide range of crack length, $c/d\cong 0.15\pm 0.05$ (Fig. 4a).

Spicule samples fractured by bending exhibited deflections of the crack path from its original direction^41–43^. In these studies, the fracture surface was generally perpendicular to the spicule longitudinal axis, but it was irregular as a result of alternating propagation and delamination cracking. In other words, when a propagating crack encountered a soft protein interface between two silica layers, it tended to deflect a certain distance, until conditions were met that enabled further propagation, and this alternation continued recursively. These experimental findings confirm the model prediction of a stepwise repetitive transition from propagation to delamination and back (Fig. 5, illustrated in Fig. 1d).

## Toward sustainable structures

Grading the thickness and stiffness of layers in a laminate has the potential of manipulating (and, therefore, optimizing) the structural resilience against a propagating crack. Such optimization may be achieved without modification of the laminate dimensions or addition of reinforcement to its structure, thus avoiding material waste and contributing to structural sustainability. To appraise the effectiveness of grading, we define the damage tolerance of a structure by its resilience in the presence of a propagating crack^17^

8 $$R=\frac{w\left(d-c\right)}{wd}=1-\frac{c}{d}$$

where $c$ is the crack depth at deflection. Thus, $R$ varies between 0 and 1. This criterion is based on the notion that when a propagating crack is deflected, the structure can still bear a load spread over its remaining cross section, $w\left(d-c\right)$, compared to the cross section of a flawless structure, $wd$ (Fig. 3). Thus, when deflection occurs while crack penetration is not too deep ($c\ll d$), the resilience is high, and vice versa.

The toughening strategies exemplified by the scorpion cuticle and the sponge spicule exhibit a clear evolutionary tradeoff: higher resilience against cracks emanating from external defects, but lower resilience against cracks emanating from internal defects. In other words, a grading trend (increasing or decreasing a property) in one direction is reversed in the opposite direction, with a likely negative impact on the resilience in that direction. Evidently, external defects are more likely to occur than internal defects, because of the exposure to the external environment and threats. This tradeoff is achieved by grading layers stiffness and thickness, without degrading the overall strength and stiffness of the structure. To obtain the same resilience without grading of properties would require different and more wasteful measures, such as stronger components, more material, and larger size. Grading optimization thus leads to higher sustainability.

**Figure 6:**
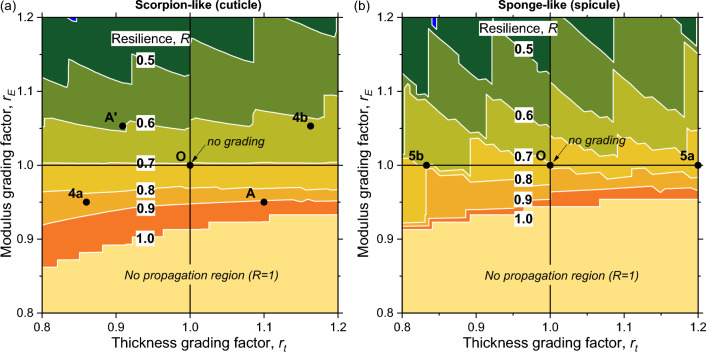
Contour maps of the structural resilience $R$ in the presence of a propagating crack, as a function of the thickness and stiffness (modulus) grading factors, ${r}_{t}={t}_{i}/{t}_{i-1}$ and ${r}_{E}={E}_{i}/{E}_{i-1}$ ($i=2..n$), respectively, for the number of layers $n=10$. (**a**) Scorpion-like cuticle structure, ${G}_{ad}/{\overline{G}}_{co}=0.11$ (Table 2). The points 4a and 4b mark the location of the cuticle examples presented in Fig. 4a,b, respectively. (**b**) Sponge-like spicule structure, ${G}_{ad}/{\overline{G}}_{co}=0.10$ (Table 2). The points 5a and 5b mark the location of the spicule examples presented in Fig. 5a,b, respectively. Points A and A’ are examples discussed in the text. The regions titled “No propagation region” designate the domain where a crack cannot propagate (the deflection condition is satisfied at $c=0$).

Of relevance to synthetic structures, a wider picture may be obtained by extending the thickness and stiffness grading range beyond that shown in the biological-like examples (Fig. 6). This is presented by mapping the resilience $R$ over the ranges $0.8\le {r}_{t}\le 1.2$ for the thickness grading factor and $0.8\le {r}_{E}\le 1.2$ for the stiffness grading factor (refer to definition of the grading factors in Table 2). A grading factor greater than 1 designates an increasing value of a property, whereas a factor smaller than 1 designates a decreasing value of a property. The colored stripes represent different ranges of resilience, as indicated on the maps, such that for each possible grading factors combination a resilience range may be obtained. These contour maps are invariant with respect to the laminate thickness $d$ and average modulus $\overline{E}$, or, in other words, they apply to any value of $d$ and $\overline{E}$ (this is derived from Eqs. (4)–(6) and (8)). Thus, the maps depend solely on the number of layers $n$ and the fracture energy ratio ${G}_{ad}/{\overline{G}}_{co}$. In that sense, these maps are universal and can be constructed for any laminate, biological-like or synthetic, given its number of layers and fracture energy ratio. The structure is a planar plate with parallel layers, with a propagating crack whose surface is perpendicular to the loading direction, as depicted in Fig. 3.

In both structural cases, the effect of modulus grading is dominant, such that, by downgrading the modulus (${r}_{E}<1$) in the crack direction, the resilience is gradually approaching the maximal possible value of $R=1$. On top of that effect, thickness upgrading in the crack direction (${r}_{t}>1$) increases the resilience further. When the modulus is not graded (${r}_{E}=1$), the contribution of thickness upgrading in the scorpion-like laminate is negligible, whereas in the sponge-like laminate it is moderate but is not monotonic with ${r}_{t}$ (that is, sometimes when ${r}_{t}$ is increased $R$ decreases; for example, moving from the left along the ${r}_{E}=1$ line, the resilience crosses from the region $R=0.7-0.8$ to the region $R=0.6-0.7$ and then back).

The locations of the biological-like examples are indicated on the maps—points 4a and 4b for the scorpion and points 5a and 5b for the sponge. Note that the points for external and internal cracks in each example are approximately diametrically opposite. The maps imply that the resilience against external cracks could hypothetically be further enhanced by thickness upgrading in the scorpion (${r}_{t}>1$) instead of downgrading (for example, moving to the right from point 4a to point A), or by adding modulus downgrading in the sponge (${r}_{E}<1$) (for example, moving from point 5a downward). This suggests that the working points chosen by nature are local optimums, governed by a resilience tradeoff between external and internal cracks, by the biological material constituents, by the structural hierarchy, and/or by other evolutionary pressures not readily observable.

Through grading, the scorpion achieves the desired resilience while reducing the amount of reinforcing material. To demonstrate this, we examine the scorpion-like example. The resilience at point 4a (Fig. 6a) is ${R}_{4\mathrm{a}}=1-14.5/100=0.86$ (Section “Toughening strategies”), whereas the resilience of a laminate with the same properties but without grading is ${R}_{\mathrm{O}}=1-29.3/100=0.71$. To reach the same resilience as in point 4a with a non-graded structure, the average cohesive fracture energy would have to be increased from $37$ J/m^2^ (Table 2) to ${G}_{co}=4\pi {G}_{ad}{R}_{4\mathrm{a}}=45.4$ J/m^2^ (Eq. (7)). Such an increase in the fracture energy would require an increase in the density of the reinforcing material (the chitin fibers) roughly by $45.4/37$=1.23, or a replacement of the reinforcing material by a tougher material; both solutions are not practical options for the scorpion.

**Figure 7:**
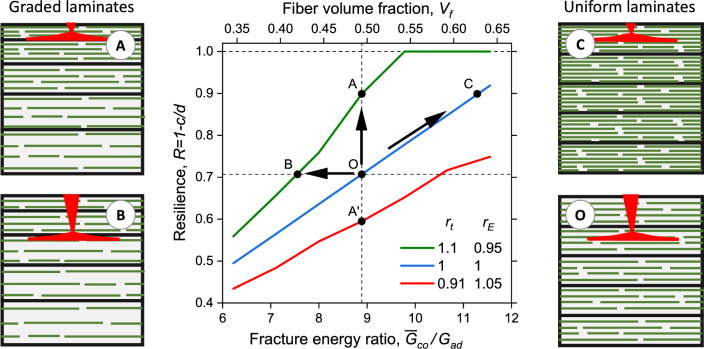
Effect of thickness and stiffness gradings on resilience and sustainability: fiber-composite example. Plot of the resilience $R$ in the presence of a propagating crack, vs. the ratio of average cohesive fracture energy ${\overline{G}}_{co}$ to adhesive fracture energy ${G}_{ad}$, for three grading combinations of thickness and stiffness, ${r}_{t}={t}_{i}/{t}_{i-1}$ and ${r}_{E}={E}_{i}/{E}_{i-1}$ ($i=2..n$). The number of layers is $n=10$. The vertical dashed line at ${\overline{G}}_{co}/{G}_{ad}\cong 8.9$ represents the conditions of the resilience map in Fig. 6a, including points A, A’ and O. The top horizontal axis represents the volume fraction ${V}_{f}$ of the reinforcing material (fibers), assumed proportional to ${\overline{G}}_{co}$. The four illustrations of uniform and graded fiber-composites correspond to points marked on the plot, and show the location of crack deflection for each case (in red). Starting from a non-graded laminate (point O), the vertical arrow shows how the resilience can be enhanced by grading (point A), whereas the horizontal arrow shows how material can be reduced without impairing the resilience (point B). Without grading, the resilience can be enhanced only by increasing material density (point C).

How can grading enhance the resilience and/or sustainability of synthetic laminates? To demonstrate the grading effect on a fiber-composite, the resilience $R$ is plotted in Fig. 7 as a function of the fracture energy ratio ${\overline{G}}_{co}$/${G}_{ad}$, with illustrations of laminate conformations. The adhesive fracture energy is assumed constant. A second horizontal axis denotes the corresponding volume fraction of the reinforcing material ${V}_{f}$. As the laminate stiffness is fairly proportional to the amount of reinforcing material^17^, and as the fracture energy in brittle materials is proportional to the stiffness^17,37^, ${\overline{G}}_{co}\left({V}_{f}\right)\sim \overline{E}\left({V}_{f}\right)\sim {V}_{f}$, and therefore the scales of ${\overline{G}}_{co}$ and ${V}_{f}$ may be assumed proportional. The resilience generally rises with an increase in the cohesive energy, or equivalently with a rise in the reinforcing material fraction, up to the limit of $R=1$. The trends in a ceramic-composite are basically similar.

A uniform laminate in this example (blue curve in Fig. 7) has a resilience of 0.71 at ${V}_{f}=0.49$ (point O), but a much higher resilience of 0.9 can be achieved by combining increasing layers thickness with decreasing modulus (green curve), without adding material (point A). To achieve this, some reinforcing material is shifted from the laminate lower part to its upper part. Alternatively, such high resilience can be achieved in a uniform laminate by increasing the material fraction by about 27% (${R}_{\mathrm{C}}/{R}_{\mathrm{O}}\cong 1.27$) (point C), but this is clearly not advantageous. When the goal is to save material without weakening the resilience, the material fraction can be reduced by about 16% by grading (point B). To achieve this, some reinforcing material is removed from the laminate lower part. The scenarios O, A, B and C are illustrated in Fig. 7, including the location of crack deflection for each case (in red). As this example is a fiber-composite, the deflection may occur at any level (see Section "Two biological structures"), at the interface between laminae (illustrated in the scenario of point A) or at a fiber-matrix interface in any location inside a lamina (illustrated in the other scenarios). Point A is also analyzed in Appendix 2: Finite element crack model. As already indicated, grading makes the laminate more resilient in the more vulnerable direction of a propagating crack, whereas the resilience in the opposite direction/grading is reduced (red curve).

The example in Fig. 7 demonstrates how fracture resilience can be controlled by merely modifying the arrangement of reinforcing material such as fibers. In particular, higher resilience can be achieved without adding more material. In terms of structural sustainability, this allows higher durability, better material efficiency and longer life cycle. Material efficiency is achieved by shifting reinforcing material from one region to another, favoring crack resistance in the more vulnerable direction. Also demonstrated is the complementary result, the potential for significant saving in reinforcing material without a degradation in resilience, which allows better material efficiency and reduction in structural weight. This has further repercussions on energy efficiency in material production and in weight-critical structures such as aircrafts.

## Conclusions

Bifurcation of propagating cracks in composite materials is an important tool for enhancing the material fracture toughness and resilience. In this study we have drawn inspiration from two very different biological structures—the scorpion’s cuticle, a composite of chitin fibers in protein, and the sponge’s spicule, a composite of silica layers with protein interfaces, both applying structural strategies that enhance toughness by deflecting cracks. This is achieved by rearrangement of material and structural components, such that both the thickness of layers and (in the cuticle) their stiffness are graded.

The condition for crack deflection was developed by applying classic fracture mechanics to laminates with variable layer thickness and stiffness, and was used in investigating two biologically inspired laminates. We found that the cuticle-like laminate deflects cracks via a decrease in the layers modulus in the crack direction, whereas the spicule-like laminate achieves this behavior by increasing the layers thickness. A wider picture is obtained by extending the thickness and stiffness grading ranges beyond those of the biological examples. We show that the grading approach can be used in synthetic laminate design to reach higher resilience (in a critically vulnerable direction) than that of a uniform laminate, without adding reinforcing material, or, alternatively, to retain the desired resilience but with significantly less material. Both grading types enhance the structural sustainability by reducing material waste and structural weight, and may potentially achieve better durability and material efficiency. The resilience mapping of the two grading trends sets a baseline for further comprehensive experimental studies, to be conducted at micro and macro scales and with different materials and structural arrangements.

In practice, implementing thickness and stiffness grading in synthetic laminates may require the use of novel approaches such as 3D printing for placing material components at desired locations in the structure in accordance with a specific grading design. This may enable creation of complex hierarchical structures, similar to those found in nature, which are non-uniform at different scales and optimized for specific design goals. Obviously, hierarchical structures go beyond the ‘simple’ model laminates investigated in this study, and are the subject of future theoretical and experimental research.

## Figures and Tables

**Figure 8:**
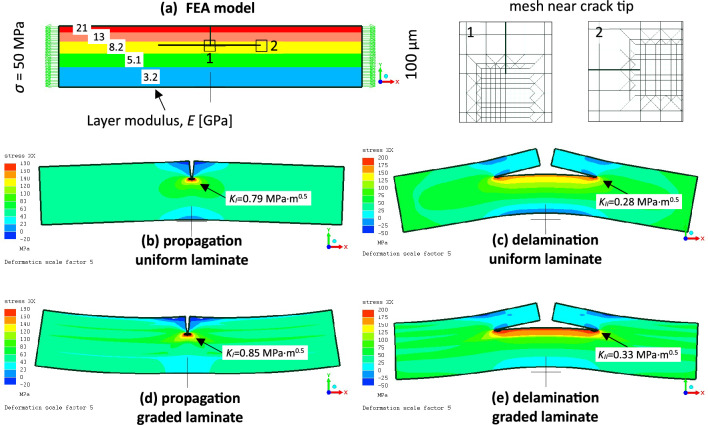
Finite element model of crack deflection. (**a**) Model definition: a 5-layer laminate, with increasing layer thickness and decreasing layer modulus (in direction $-y$), subjected to tensile loading in the x-direction. The induced propagation and delamination cracks, and their fine element meshing, are shown. (**b**–**e**) Deformation (× 5) and stress fields in uniform and graded laminates, with propagation and delamination cracks. The derived stress intensity factors ${K}_{I}$ and ${K}_{II}$ for mode I (propagation) and mode II (delamination) are indicated for each case.

## Data Availability

The authors declare that all data generated or analyzed during this study are included in this published article.

## Appendix 1: Deflection condition in bending

The analysis in Section “Crack deflection condition” applies to tensile loading. Herein, we expand the analysis to bending loading of the same structure, namely of a planar laminate with a penetrating crack whose surface is perpendicular to the loading direction (Fig. 3). An approximation can be obtained by analogy, where the cohesive and adhesive flexural (bending) stresses are expressed by Eqs. (2) and (3), respectively, replacing the tensile moduli by flexural (bending) moduli. Thus:

$${\widetilde{\sigma }}_{co}=\sqrt{\frac{{G}_{co}\widetilde{E}}{\pi c}}\sqrt{\frac{\widetilde{E}}{{\widetilde{E}}_{1}}}$$

9 $${\widetilde{\sigma }}_{ad}=\sqrt{\frac{{2G}_{ad}\widetilde{E}}{c}\left(1-\frac{c}{d}\right)}\sqrt{\frac{{\widetilde{E}}_{2}}{{\widetilde{E}}_{1}}}$$

$\widetilde{\sigma }$ denotes the flexural stress, or the stress at the laminate boundary: $\widetilde{\sigma }=\frac{Md}{2I}$ where $M$ is the bending moment and $I$ is the laminate moment of inertia. $\widetilde{E}$ denotes the flexural modulus, or the elastic resistance to bending: $\widetilde{E}=\frac{K}{I}$ where $K$ is the laminate flexural stiffness. The deflection condition is given by (Eq. (4))﻿:

10 $$\frac{{G}_{ad}}{{G}_{co}}<\frac{1}{4\pi \left(1-c/d\right)}\frac{\widetilde{E}}{{\widetilde{E}}_{2}} , \quad \quad \quad c\ll d$$

The flexural moduli are defined by:

11 $${\widetilde{E}}_{i}=\frac{{\int }_{a}^{b}{z}^{2}E\left(z\right)dz}{{\int }_{a}^{b}{z}^{2}dz}$$

where the nominator is the flexural stiffness and the denominator is the moment of inertia, both per laminate unit width. The integration limits $\left(a,b\right)$ are $\left(-\frac{d}{2},\frac{d}{2}\right)$ for $\widetilde{E}$, $\left(\frac{d}{2}-c,\frac{d}{2}\right)$ for ${\widetilde{E}}_{1}$, and $\left(-\frac{d}{2},\frac{d}{2}-c\right)$ for ${\widetilde{E}}_{2}$. The origin of $z$ is at the laminate midplane, and $E\left(z\right)$ is the tensile modulus at location $z$. Equation (11) can be expressed in discrete form as in Eq. (5).

Generally, the crack deflection trend is similar to that of tensile loading, because the flexural modulus ${\widetilde{E}}_{2}$ tends to be high in regions where the average tensile modulus ${\overline{E}}_{2}$ is high, and vice versa. Specifically, the flexural modulus ${\widetilde{E}}_{2}$ is lower for a laminate with decreasing modulus, compared to a laminate with increasing modulus, making the right side of the inequality in Eq. (10) larger, consequently increasing the likelihood of deflection and the resilience.

## Appendix 2: Finite element crack model

The analysis in Section “Crack deflection condition” uses classic fracture mechanics, balancing the elastic energy release rates during crack propagation against the material cohesive and adhesive fracture energies. Herein, we provide further insight by examining the deformation, stress fields, and stress intensity factors in graded laminates using finite element analysis (FEA) (Fig. 8). The model structure is a laminate with increasing layer thickness and decreasing layer modulus (Fig. 8a), similar to case A in Fig. 7. Crack propagation and deflection FEA simulation will be the subject of further study.

The solver used in the analysis is Mecway v13, in Static 3D mode. The plate dimensions are L500 × H100 × T2.5 μm. The element type is solid Hex8 (8-node box), with solid Pyr5 (5-node pyramid) in regions of meshing density transition. The number of elements is 14,720–16,442 and the number of nodes is 22,729–24,311, depending on the specific case. The elements density near a crack tip is 4 times higher in each direction than in the regions far from the tip. The material is linear elastic isotropic without failure criteria, with Poisson’s ratio 0.33, and with modulus of 8 GPa for the uniform cases and varying moduli as shown in Fig. 8a for the graded cases. The layers are firmly connected to each other via shared nodes. Uniform stress of + 50 MPa is applied to the laminate right face and − 50 MPa to its left face. The elastic model allows observing the stress fields near a crack tip, specifically the stress intensity factors ${K}_{I}$ and ${K}_{II}$, in order to predict the effect of thickness and stiffness grading on crack deflection likelihood.

Fracture mechanics premises are generally supported by the model in both uniform and graded formations. In a propagating crack (Fig. 8b,d), the stress above the crack tip is low (blue triangular region), as it was released when the crack was introduced; the released energy may cause further progress of the crack if it overcomes the material cohesive fracture energy. In a delamination crack (Fig. 8c,e), the stress above the crack is low (blue rectangular regions), and its released energy is partially gained by the region below the crack (green region); the net energy release may cause further delamination progress if it overcomes the material adhesive fracture energy.

The grading of thickness and stiffness changes the deformation and stress fields significantly. In a propagating crack (Fig. 8d), the deformation is convex, compared to concave in a uniform laminate; the stress field is similar in both cases, but the opening (mode I) stress intensity factor ${K}_{I}\cong \underset{r\to 0}{\mathrm{lim}}\left({\sigma }_{xx}\sqrt{2\pi r}\right)$ (stress matching method, where $r$ is the distance ahead from the crack tip)^44^ is higher in a graded laminate by 8% (see ${K}_{I}$ values in Fig. 8b,d), indicating higher tendency to propagate further. However, this stress concentration vanishes in the presence of a delamination crack, and is replaced by stress concentration at the delamination tips; the stress levels below the delamination crack and at its tips are higher in the graded laminate (red and black regions); in this case in-plane shear (mode II) is the dominant fracture mode, and the stress intensity factor ${K}_{II}\cong \underset{r\to 0}{\mathrm{lim}}\left({\tau }_{xy}\sqrt{2\pi r}\right)$ is higher in a graded laminate by 18% (see ${K}_{II}$ values in Fig. 8c,e), indicating higher tendency to propagate further; note also the different deformation in the delaminating graded structure. These stress intensity factors imply that the likelihood of delamination due to grading is higher than that of propagation (see the discussion in Section “Crack deflection condition”), indicating higher tendency for crack deflection, resulting in higher resilience as predicted.

In the extreme case when $c\to d$, the FEA shows that the stress intensity factor rises faster in a propagating crack than in a delamination crack. For example, in a uniform laminate with a crack $c=0.9d=90$ μm, ${K}_{I}$ increases by a factor of 38 with respect to the crack in Fig. 8, whereas ${K}_{II}$ increases by a smaller factor of 32. Thus, the tendency to deflect decreases in very deep cracks, and as $c$ approaches $d$ deflection becomes more unlikely.
